# Exploring the complexities of plant UV responses; distinct effects of UV-A and UV-B wavelengths on Arabidopsis rosette morphology

**DOI:** 10.1007/s43630-024-00591-w

**Published:** 2024-05-12

**Authors:** Natalie Cunningham, Gaia Crestani, Kristóf Csepregi, Neil E. Coughlan, Marcel A. K. Jansen

**Affiliations:** 1 School of Biological, Earth and Environmental Sciences, Environmental Research Institute, University College Cork, North Mall, Cork, Ireland; 2 Department of Plant Biology, Institute of Biology, University of Pécs, Ifjúság u. 6, 7624 Pecs, Hungary

**Keywords:** UV-A, UV-B, Morphology, Arabidopsis, Acclimation, UVR8

## Abstract

UV-B radiation can substantially impact plant growth. To study UV-B effects, broadband UV-B tubes are commonly used. Apart from UV-B, such tubes also emit UV-A wavelengths. This study aimed to distinguish effects of different UV-B intensities on *Arabidopsis thaliana* wildtype and UVR8 mutant rosette morphology, from those by accompanying UV-A. UV-A promotes leaf-blade expansion along the proximal–distal, but not the medio-lateral, axis. Consequent increases in blade length: width ratio are associated with increased light capture. However, petiole length is not affected by UV-A exposure. This scenario is distinct from the shade avoidance driven by low red to far-red ratios, whereby leaf blade elongation is impeded but petiole elongation is promoted. Thus, the UV-A mediated elongation response is phenotypically distinct from classical shade avoidance. UV-B exerts inhibitory effects on petiole length, blade length and leaf area, and these effects are mediated by UVR8. Thus, UV-B antagonises aspects of both UV-A mediated elongation and classical shade avoidance. Indeed, this study shows that accompanying UV-A wavelengths can mask effects of UV-B. This may lead to potential underestimates of the magnitude of the UV-B induced morphological response using broadband UV-B tubes.

**Graphical abstract:**

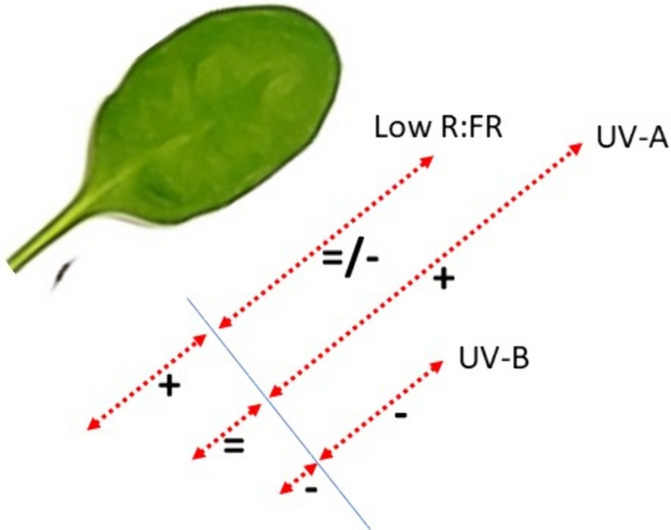

## Introduction

Effects of light on plants are multifaceted. Light is an essential source of energy driving photosynthesis but can also negatively impact plants by causing photoinhibitory or UV-radiation damage. Further, light can steer plant growth and development directly through photoreceptors or photosensitiser [1, 2] or indirectly through effects on other organisms such as pollinators and frugivores [3]. These light-dependent processes are described by action spectra, which may differ considerably in terms of the specific wavelengths that drive a particular process. Thus, depending on the quality and quantity of light, distinct physiological and developmental responses will be triggered. Effects of ultraviolet B radiation (UV-B, 280–315 nm) on plants have been extensively studied [4, 5]. Two major types of UV-B driven processes can be distinguished, those mediated through the UV-B photoreceptor UVR8 and those that are associated with various forms of damage and/or plant stress [6]. Environmentally-relevant doses of UV-B radiation are not necessarily considered a plant stressor, but acting together with other abiotic factors can induce stress, either directly or indirectly via induced reactive oxygen species (ROS) [6]. There is a further distinction between UV-B and UV-A (315–400 nm) driven processes [7], although it has been argued that from a plant biological perspective the distinction between the UV-B and UV-A wavelength zones is not useful [8]. Indeed, the UVR8 action spectrum, although displaying a prominent monomer formation peak at around 280 nm under in vitro conditions nm [9], does mediate perception of wavelengths well into the UV-A part of the spectrum, and as high as 350 nm [8]. In the UV-A part of the spectrum, further photoreceptors are active, especially cryptochromes which are known to co-regulate gene-expression in association with UVR8 [8, 10]. Given both spectral overlap and interactive effects between photoreceptors, it can in practice be difficult to study UV-B radiation responses in the absence of background UV-A radiation effects. This is particularly true given the widespread use of broad band UV sources which emit both UV-B and UV-A wavelengths, and whereby any increase in UV-B is accompanied by an increase in UV-A [11].

A broad range of UV-B effects has been detailed in the literature [5, 12–14]. Some of these UV effects are caused by exposure to high doses of UV-B and are associated with oxidative stress, inactivation of photosynthetic activities and/or DNA damage, i.e. plant stress [6, 15]. In contrast, lower UV-B doses trigger a range of regulatory responses including protective responses such as increases in antioxidant status, photorepair capacity and contents of UV-absorbing pigments [16]. These regulatory responses are closely associated with the photoreceptor UVR8 [17]. One UV response that may either be UV-B stress associated (i.e. stress-induced morphogenic response, cf. [18] or a regulatory response (i.e. UVR8 photoreceptor mediated) is the UV-B mediated change in plant morphology. This response typically comprises development of a more compact plant with shorter stems, increased axillary branching or tillering, thicker leaves, shorter petioles, altered root:shoot ratio and inflorescence structures [19]. The functional role of these UV-B induced morphological changes remains largely unproven. Increased self-shading of leaves due to reduced elongation of stems and petioles has been hypothesised to contribute to diminished UV exposure [12], although it remains to be seen whether this is correct given the high percentage of scattered UV-radiation within a canopy [20]. Conversely, where UV-B induced morphological responses are stress associated (i.e. linked to ROS, NO and oxidative stress cell cycle checkpoints) [19] the UV-B phenotype might not necessarily have an adaptive role. Interestingly, UV-A wavelengths also affect plant morphology, although effects are quite different with UV-A exposure [21]. UV-A, amongst others, stimulates leaf expansion [7]. Thus, the question arises whether UV-A wavelengths can moderate, or even completely mask, UV-B responses when plants are exposed to a mixture of wavelengths, as is the case in natural sunlight. The answer will ultimately depend on used UV intensities and/or doses. Unfortunately, knowledge of dose–response curves for plant UV-effects is scarce, and almost completely lacking in the case of plant morphological UV responses [19]. Yet, in the few cases where responses to multiple UV intensity levels were determined, dose–response curves were complex. For example, in a classic study, Brodführer [22] observed that low UV doses induced different effects on Arabidopsis morphology than higher doses (also see [19]). Particularly inflorescence branching displayed a bell-shaped response curve with maximal branching at intermediate intensities. Similarly, *Silene noctiflora* leaf number and specific leaf weight decrease under low UV intensities but increase under higher levels [23]. However, other studies have not confirmed such a bell-shaped dose–response [24].

The aim of the current study is to differentiate effects of different UV-B intensities on the rosette morphology of *Arabidopsis thaliana* wildtype and UVR8 mutant plants, from those caused by accompanying UV-A. It is hypothesized that UV-A radiation emitted by commonly used broad-band UV-B tubes will counter UV-B induced effects, and therefore lead to an underestimate of actual UV-B effects.

## Materials and methods

### Plant material

*Arabidopsis thaliana* wild type (Col-0 WT) and *uvr8-6* [25] genotypes were utilised in this study. *Uvr8-6* seeds were kindly donated by Professor Ulm of the University of Geneva, Switzerland. Seeds were plated on moistened filter paper in petri dishes and stored at 4 °C for 72 h. Stratified seeds were then sown directly onto moist jiffy plugs (Deker Horticulture, Co. Meath, Ireland), and placed in plastic containers. Containers were covered with cling film and placed under cool white Photosynthetically Active Radiation (PAR) (MASTER TL5 HE 28W/840 SLV/40) light in a controlled plant growth room. PAR intensity was 60–80 µmol m^−2^ s^−1^ and plants were subject to a 14-h light/10-h dark photoperiod. To avoid excessive plant elongation, the red light intensity was at least 5-times greater than the far-red intensity. Temperature and relative humidity were set to 22 °C and 60% respectively. Once germination had occurred and seedlings were established (5 days), the cling film was removed. Excess plantlets were removed using tweezers, to leave a total of one plant per jiffy plug. Plants continued to grow for a total of 15 days until the 1.04 Boyes growth stage [26] and were watered twice weekly.

### UV exposure conditions

Following the growth period, plants were transferred to a self-contained UV growth chamber in which the PAR intensity was set at 80–90 µmol m^−2^ s^−1^ (MASTER TL5 HE 28W/840 SLV/40) and the photoperiod was16-h light/8-h dark. To avoid any impact of the small change in PAR intensity, plants were acclimated for one day under the new growth conditions, and prior to UV exposure. On the next day in the UV chamber, plants were primed with UV for 30 min. Thereafter, plants were exposed to UV for 2 h at noon, for a total of 7 days. UV radiation was emitted by two broadband UV-B tubes (TL40W/12 Philips, Germany), which emit UV-B, as well as UV-C, UV-A, and some PAR radiation. To block UV-C radiation, TL tubes were loosely wrapped in a single layer of cellulose acetate (CA) (95 μm thickness; Kunststoff-Folien-Vertrieb GmbH, Hamburg, Germany). To remove ≥ 95% UV-B wavelengths from the spectrum, a UV-B blocking filter (Mylar − 125 µm thickness, Tocana Ltd., Ballymount, Ireland). Mylar (or polyester) filters effectively block UV-B radiation, as well as shorter UV-A wavelengths. Thus, the UV treatments are categorised as either UV-A/B enriched, comprising PAR, UV-B as well as accompanying UV-A radiation emitted by the UV-B tube, or as UV-A enriched, comprising PAR and UV-A. Both CA and Mylar filters were placed above the plants. For the “0 UV” treatment, plants filters were also used in the absence of any UV. The used UV filters had minimal (< 2%) impact on the PAR intensity and the spectral distribution experienced by the plants. Cellulose acetate filters were replaced after 20 h of UV exposure, when transmission properties started to change.

Due to capacity issues, plants were divided into two groups, plants exposed to either UV-A enriched or UV-A/B enriched radiation. Both groups included “0 UV” controls for each genotype/treatment combination, such that each group of UV-A enriched or UV-A/B enriched treatments has its own “internal” control. Minor differences between “0 UV” controls relate to biological variability in used plant material.

The UV intensity was adjusted using a dimmable ballast (Sylvania-Biosystems, Wageningen, The Netherlands). Experiments comprised exposure to four UV intensities described as zero, low, moderate, and high UV (Table 1). Absolute irradiance was measured using an optical fiber diode-array spectroradiometer equipped with a cosine corrector (Flame-S, Ocean Optics, Duiven, The Netherland), for every intensity and filter combination using the manufacturers software, Oceanview (version 1.6.7). UV intensity was expressed in µW cm^−2^, as well as µmol m^−2^ s^−1^. The biological effective dose was calculated according to Flint and Caldwell [27] (Table 1). Each experiment was independently repeated a total of three times for each treatment, genotype, and intensity combination.

**Table 1:** UV conditions including irradiance (µW/cm^2^), biological dose (kJ/m^2^), and ratios under UV-A/UV-B enriched treatments

	UV-B irradiance (µW/cm^2^)	UV-B irradiance (µmol/m^2^ s)	UV-B biological dose (kJ/m^2^)	UV-A irradiance (µW/cm^2^)	UV-B irradiance (µmol/m^2^ s)	UV-A biological dose (kJ/m^2^)	Ratio of UV-B: UV-A irradiance	Ratio of UV-B:UV-A biological dose
**UV-A enriched**								
Low	BDL		N/A	11.695	0.32	0.037	N/A	N/A
Moderate	BDL		N/A	45.209	1.27	0.142	N/A	N/A
High	BDL		N/A	156.94	4.33	0.489	N/A	N/A
**UV-A/B enriched**								
Low	11.408	0.30	0.552	19.941	0.55	0.067	0.572	8.239
Moderate	43.443	1.17	1.976	75.102	2.07	0.257	0.578	7.689
High	154.36	4.06	6.779	268.34	7.40	0.901	0.575	7.524

Biologically effective doses were calculated according to Flint and Caldwell [27]. Values shown for low, moderate, and high irradiances. BDL was used to indicate where intensity was below the detection

### Morphological analysis

Destructive morphological assays were performed on the 7th day of UV treatment, promptly after the cessation of the UV radiation. Firstly, the direction of the growth spiral was identified, and leaves were numbered, beginning with the oldest first true leaf and increasing with developmental order. Cotyledons were not considered in this process.

Three plants per treatment and genotype were then dissected as part of each replicate. Only leaves which had petioles > 2 mm in length were sampled, while small, newly emerging and leaves and leaf primordia were excluded from the analysis. Consequently, a total of eight leaves were included in analyses. Petiole length, leaf blade length, leaf width and leaf area were quantified from photos using ImageJ 1.53c software [28]. For statistical analysis, leaves were grouped in three categories (developmental phases) according to their ontogeny (Table 2). For morphological analysis three plants were dissected per experiment (n = 9).

**Table 2:** Description of rosette developmental stages

Rosette developmental phase (RD)	Description of growth stage	Leaf number
RD 1	Leaves fully formed and outgrown prior to UV exposure	1, 2, 3
RD 2	Juvenile leaves (not fully expanded) as well as leaf primordia visible prior to UV exposure	4, 5, 6
RD 3	Leaves not yet visible at the start of the UV exposure experiment, but that expanded during UV exposure	7, 8
Depiction of leaves as observed following 7 days of UV-treatment		

### Chlorophyll *a* fluorometry

Photosynthetic efficiencies were measured using a pulse amplitude modulated imaging chlorophyll fluorometer (WALZ, Effeltrich, Germany). Plants were dark adapted for 20 min before measurement of ground (F0) and maximal (Fm) fluorescence, which in turn facilitated calculation of the maximum quantum yield of photosystem II (PSII) (Fv/Fm). Thereafter a background actinic light of 80 μmol m^−2^ s^−1^ was applied for five minutes to reach a steady-state. A saturating pulse of photosynthetically active light was applied to measure both Fm’ and Ft. The effective quantum yield (YII) was calculated according to Murchie and Lawson [29]. Three plants (n = 3) were measured at the end of each seven-day UV exposure, and each experiment was independently repeated three times (n = 9). For analysis of UV effects, effects on leaves 1–3, leaves 4–6 and leaves 7–9 were pooled to give three developmental classes.

### Assessment of antioxidant capacity

Antioxidant capacities were measured using the both Folin–Ciocâlteu and Ferric-Reducing Antioxidant Power assays (FRAP). FRAP is particularly responsive to changes in flavanol content while Folin–Ciocâlteu is more sensitive to phenolic acids [30]. Thus, together, these assays provide a comprehensive insight into UV induced antioxidant capacities.

The pooled leaves of individual rosettes, comprising 50–60 mg of fresh tissue, were placed in 2 mL tubes and ground in liquid nitrogen. Samples were extracted using 600 µL of 70% ethanol, vortexed briefly and placed in an iced sonicator for 20 min. Samples were subsequently centrifuged for 10 min at 12,000 rpm and the supernatant was collected. The pellet was resuspended in a further 400 µL of 70% ethanol, vortexed, sonicated on ice, centrifuged for 10 min at 12,000 rpm and the supernatant was collected. Both supernatants were combined to give a final volume of 1 mL. For antioxidant assays, all true leaves of three individual plants were analysed (n = 9).

#### Folin–Ciocâlteu reactivity

The method is based on Singleton and Rossi [31] with modifications as detailed by Csepregi et al. [30]. An aliquot of 100 µL of plant extract was mixed with Folin–Ciocâlteu Reagent (FCR) (diluted with distilled water 1:10) and left at room temperature for five minutes. Subsequently, 500 µL NaCO_3_ 6% (w/v) was added, and samples were incubated in darkness for 90 min. Absorbance was recorded at 765 nm using a spectrophotometer (Thermo Scientific™ GENESYS™ 50 UV–Vis Spectrophotometer). Gallic acid was used to build a calibration curve and FCR values of plant samples were reported in gallic acid equivalents.

#### Ferric-reducing antioxidant power

The Ferric Reducing Antioxidant Power (FRAP) is measured based on the iron-reducing ability of the antioxidants in the sample [32]. Iron(III) is reduced to Iron(II) by metabolites with antioxidant properties, and this is accompanied by the development of a blue colour. The assay followed a modified version of Csepregi et al. [16]. Firstly, FRAP reagent was prepared by combining 12.5 mL of acetate buffer (300 mM, pH 3.6), 1.25 mL 2,4,6-tripyridin-2-yl-1,3,5-triazine (TPTZ) (10 mM TPTZ in 40 mM HCl (37%)) and 1.25 mL of FeCl_3_ (20 mM in distilled water). Thereafter, 950 µL of FRAP reagent was mixed with 50 µL plant extract and incubated at room temperature, for 30 min. Samples were very gently vortexed every 10 min during this period. Absorbance was measured at 592 nm using a spectrophotometer. Ascorbic acid was used to build a calibration curve and FRAP values of plant samples were expressed as ascorbic acid equivalents.

### Statistical analysis

Statistical analyses were performed using R software (R Core Team 2021; R 4.1.2). Normality and variance assumptions were tested using Shapiro–Wilk tests (*p* > 0.05) and Levene’s tests (*p* > 0.05), respectively. Linear regression was used to analyze normally distributed and homoscedastic residuals. Logistic regression in the form of generalised linear models (GLM: car) was used if assumptions of normality and/or equal variance of residuals were not met. A stepwise depletion approach was used to remove non-significant terms, while overall model significance was determined using likelihood ratio tests in all cases (lmtest). As intensity-driven trends for a specific genotype (Col-0 WT vs *uvr8-6*) exposed to a specific type of UV-radiation treatment (UV-A enriched or UV-A/B enriched) can be masked in GLM, due to the known biological response for the genetically engineered mutant, a further one-way ANOVA, or corresponding nonparametric Kruskal–Wallis H test, was used to explore significant effects of intensities within each separate genotype or treatment group, in those cases where GLM had shown a statistically significant effect of intensity. Each group consisted of four intensity levels. Assumptions were tested as specified with ANOVA or Kruskal–Wallis H tests performed using IBM^®^ SPSS^®^ Statistics Version 28.0 software.

## Results

*Arabidopsis thaliana* wild-type (Col-0) and a UVR8 photoceptor mutant (*uvr8-6*) were grown for 7 days under a broad band UV-source using either Mylar (UV-A enriched treatment) or cellulose acetate filters (UV-A/B enriched treatment). A total of four UV intensities were used namely: zero UV (PAR only), low UV, moderate UV, and high UV (Table 1). Morphological parameters, chlorophyll *a* fluorescence, and antioxidant capacity were measured on the final day of UV exposure.

### Morphological responses to different UV treatments

#### Leaf blade length

**Fig. 1:**
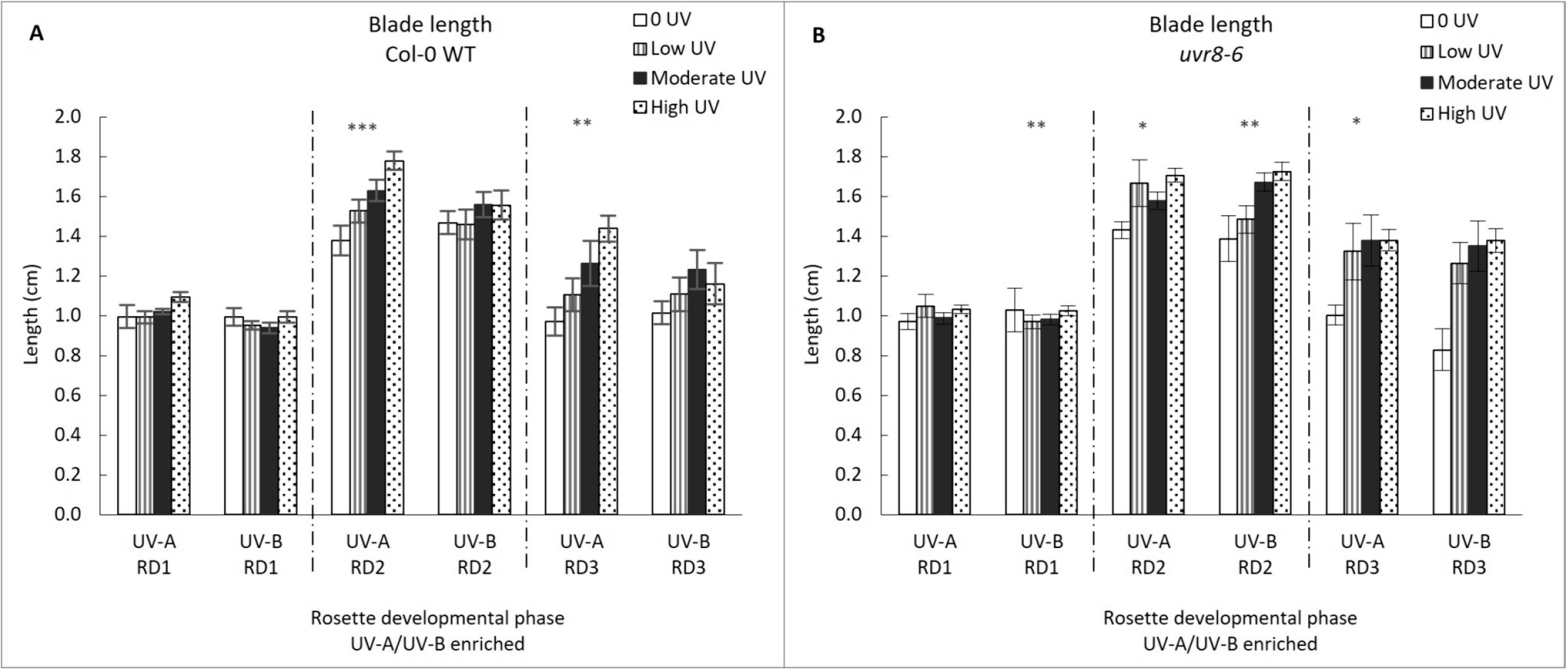
Leaf blade length (cm) in Col-0 WT (**A**) and *uvr8-6* (**B**), across the three rosette developmental phases (RD1, RD2, RD3) and two treatments (UV-A/B and UV-A enriched). Bars (with standard error) represent mean blade length. Bars represent different UV intensities: white bar = “0 UV”, striped bar = “Low UV”, black bar = “Moderate UV”, dotted bar = “High UV”. Blade length was significantly affected by increasing UV-A intensity for Col-0 across RD2 (ANOVA, p < 0.001) and RD3 (ANOVA, p = 0.003) and *uvr8-6* across RD2 (K Wallis, p = 0.012) and RD3 (K Wallis, p = 0.038). UV-A/B had a significant effect on blade length for *uvr8-6* plants in both RD1 (K Wallis, p = 0.009) and RD2 (ANOVA, p = 0.002). Asterisks indicate a significant difference between 0 UV and low, moderate and high intensities with p < 0.05 (*), p < 0.01 (**), or p ≤ 0.001 (***)

Blade length of Col-0 and *uvr8-6* plants is substantially affected by different UV treatments (UV-A/B and UV-A enriched radiation) and varying intensity levels (Fig. 1). Responses vary depending on the developmental stage. Older leaves (RD1) show no statistically significant responses to UV exposure and UV intensity and blade length does not vary with genotype (GLM: χ^2^ = 13.212, df = 15; p > 0.05). However, blade length responds significantly to treatment in developmental phase 2 (GLM: χ^2^ = 33.073, df = 3; p < 0.001), intensity was the key effects driver (p < 0.0001), while genotype and UV treatment had no effect (both p > 0.05) with no interactive effects detected (all p > 0.05). For developmental phase 2, one-way ANOVA shows a significant increase in WT blade length with increasing intensity in the UV-A enriched treatment (p < 0.0001; Fig. 1). There is no significant change in blade length with intensity in the UV-A/B enriched treatment. In contrast, blade length of the *uvr8-6* mutant increased significantly as a function of intensity across both UV-A and UV-A/B enriched treatments (p < 0.05 and p < 0.01, respectively).

In developmental phase 3, blade length also responds significantly to treatment (GLM: χ^2^ = 36.427, df = 3; p < 0.001). Only intensity was a significant driver of differences in RD3 blade length (p < 0.0001), all other factors and potential interactive effects were not statistically apparent (all p > 0.05). One-way ANOVA shows a significant increase in WT blade length in the UV-A enriched treatment (p < 0.01), but no significant change in the UV-A/B enriched treatment, with increasing intensity. In contrast, *uvr8-6* blade length increased significantly as a function of intensity across both UV-A and UV-A/B treatments (p = 0.05 and p = 0.01, respectively).

#### Leaf blade width

**Fig. 2:**
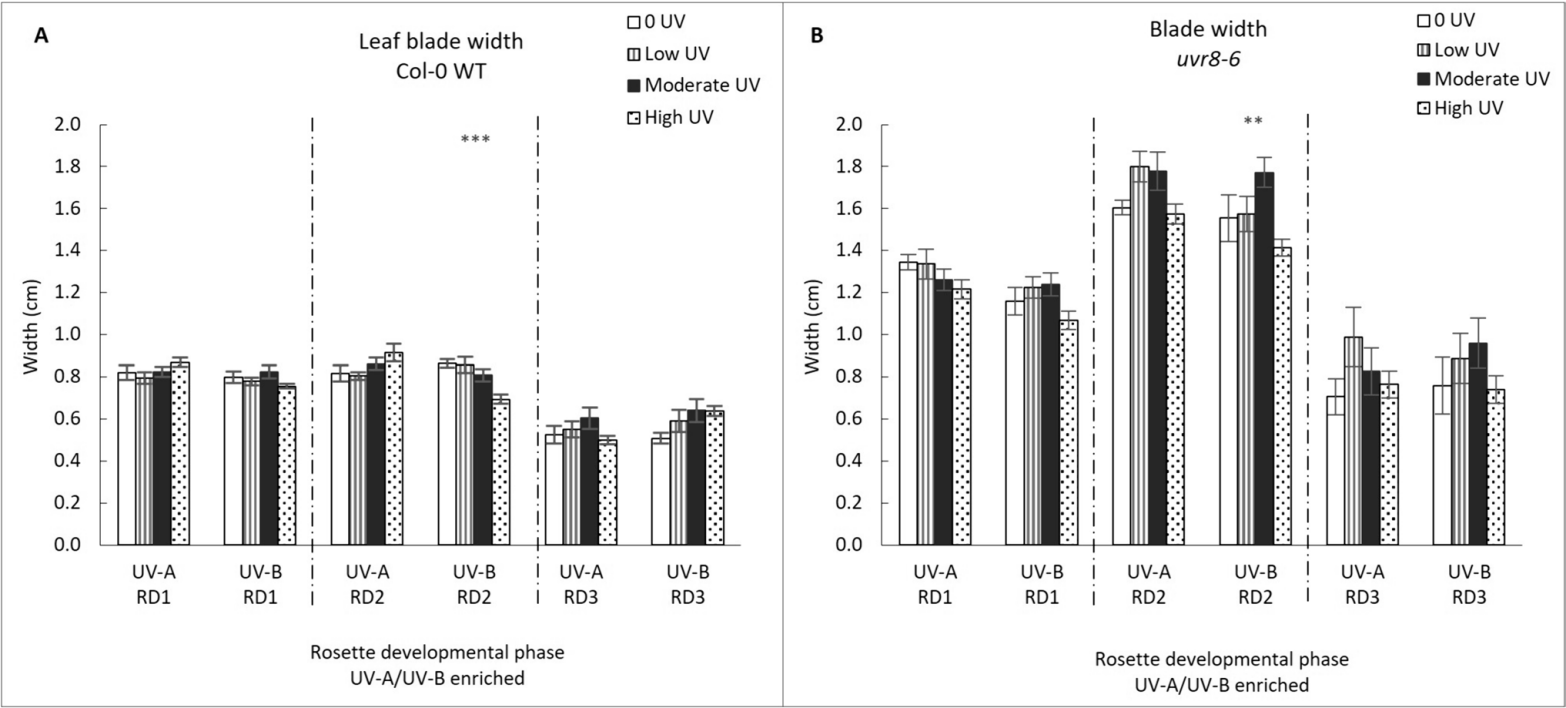
Leaf blade width (cm) in Col-0 WT (**A**) and *uvr8-6* (**B**), across the three rosette developmental phases (RD1, RD2, RD3) and two treatments (UV-A/B or UV-A enriched). Bar charts (with standard error) represent mean blade width. Bars represent different UV intensities: white bar = “0 UV”, striped bar = “Low UV”, black bar = “Moderate UV”, dotted bar = “High UV”. **A**, **B** Blade width was significantly affected under the UV-A/B enriched treatment only in RD2 for both Col-0 (ANOVA, p < 0.001) and *uvr8-6* (Welch ANOVA, p = 0.005). Asterisks indicate a significant difference between 0 UV and low, moderate and high intensities with p < 0.05 (*), p < 0.01 (**), or p ≤ 0.001 (***)

Blade width of Col-0 and *uvr8-6* plants was moderately affected by UV treatment (UV-A/B and UV-A enriched and varying intensities) and genotype (Fig. 2). Overall, it was found that leaves of *uvr8-6* are wider than those of Col-0 across all stages of development, treatments, and intensities. Blade width responds significantly to treatment in developmental phase 2 (GLM: χ^2^ = 331.42, df = 15; p = < 0.0001), with genotype (p < 0.0001), intensity (p < 0.001) and UV treatment (p < 0.01) all having a significant effect on blade width. Further significant interactive effects were detected between intensity and UV treatments (p < 0.05), as well as among genotype, intensity and UV treatment (p < 0.01). For developmental phase 2, one-way ANOVA shows a significant decrease in blade width with increasing intensity in the UV-A/B treatment, both in the WT and *uvr8-6* (p < 0.001 and p < 0.01, respectively). No significant changes in blade width were noted for UV-A enriched treatment.

Blade width responds significantly to treatment in developmental phase 3 (GLM: χ^2^ = 49.676, df = 4; p = < 0.0001). Overall, in phase 3 genotype (p < 0.0001) and level of intensity (p = 0.05) play a significant role in the width of leaves, while UV treatment and all potential interactions had no statistically apparent effects (all p > 0.05). However, ANOVA did not reveal any specific intensity dependent changes across the UV-A/B and UV-A enriched treatments of WT Arabidopsis, and the *uvr8-6* mutant.

#### Leaf blade area

**Fig. 3:**
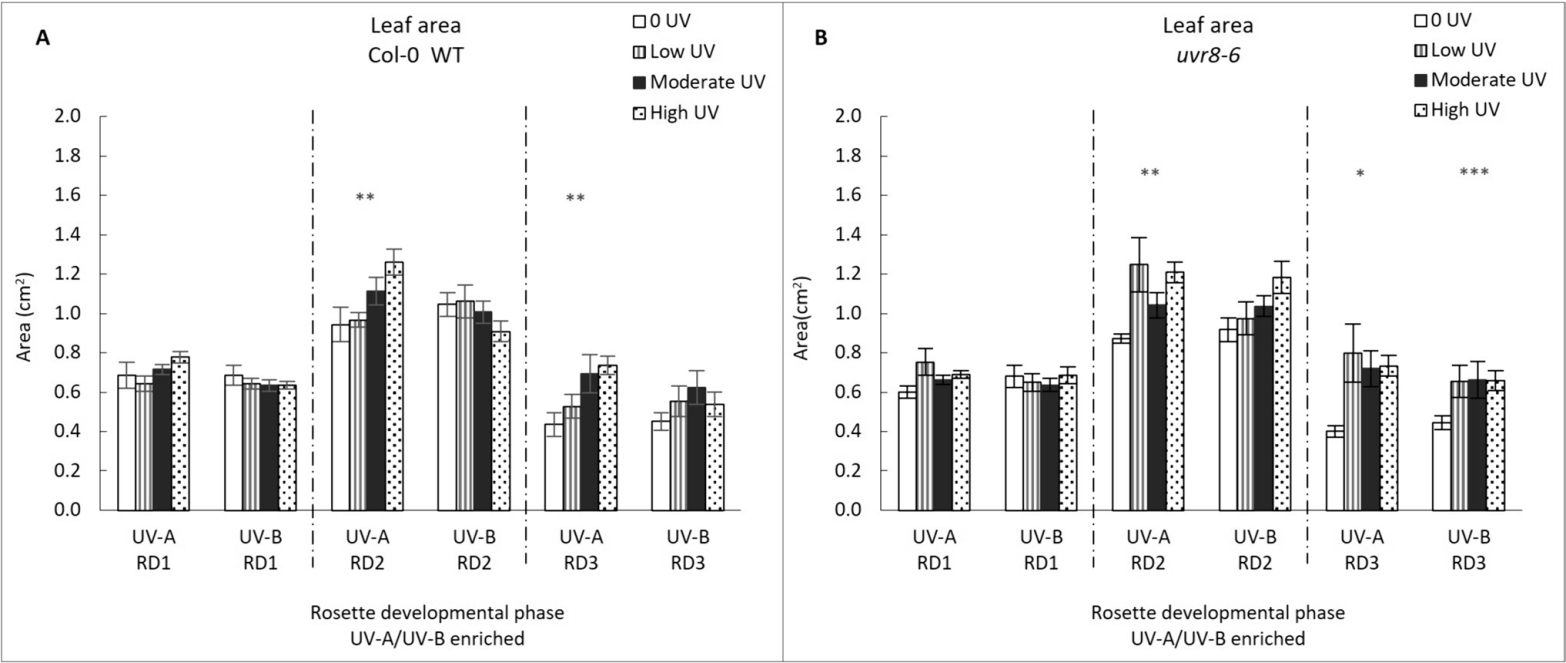
Leaf area (cm^2^) in Col-0 (**A**) and *uvr8-6* (**B**), across the three rosette developmental phases (RD1, RD2, RD3) and two treatments (UV-A/B or UV-A enriched). Bar charts (with standard error) represent mean leaf area. Bars represent different UV intensities: white bar = “0 UV”, striped bar = “Low UV”, black bar = “Moderate UV”, dotted bar = “High UV”. **A** Leaf area was affected under UV-A for Col-0 across RD2 (ANOVA, p = 0.007) and RD3 (Welch ANOVA, p = 0.006). **B** In *uvr8-6* plants, leaf area was significantly affected by UV-A in RD2 and RD3 (K Wallis, p = 0.003; p = 0.015) while UV-A/B treatment was significant for RD3 (Welch ANOVA, p = 0.009). Asterisks indicate a significant difference between 0 UV and low, moderate and high intensities with p < 0.05 (*), p < 0.01 (**), or p ≤ 0.001 (***)

UV or genotype mediated changes in blade length and width can potentially affect the total blade area. The leaf area of older leaves (RD1) shows no statistically significant response to UV exposure and UV intensity and leaf area does not vary with genotype either (Fig. 3). Statistically significant effects on leaf area were noted for phases 2 (GLM: χ^2^ = 44.733, df = 15; p = < 0.0001) and 3 (GLM: χ^2^ = 29.382, df = 3; p = < 0.0001). In phase 2, leaf area was primarily affected by intensity (p < 0.001) however, an interactive effect among genotype, intensity and UV treatment also plays a role (p < 0.01). One-way ANOVA shows a significant increase in RD2 blade area with increasing intensity in the UV-A treatment in both WT and *uvr8-6* (p = 0.007 and p = 0.003, respectively). A non-significant decrease in leaf area with increasing intensity is noted for the WT exposed to the UV-A/B treatment, as is a non-significant increase in leaf are for *uvr8-6*. In developmental phase 3, the UV-A enriched treatment induces significant increases in leaf area across both genotypes (p = 0.006 and p = 0.015, respectively for WT and *uvr8-6*), while the UV-A/B enriched treatment triggered an increase in leaf area in *uvr8-6* only (p = 0.009).

#### Petiole length

**Fig. 4:**
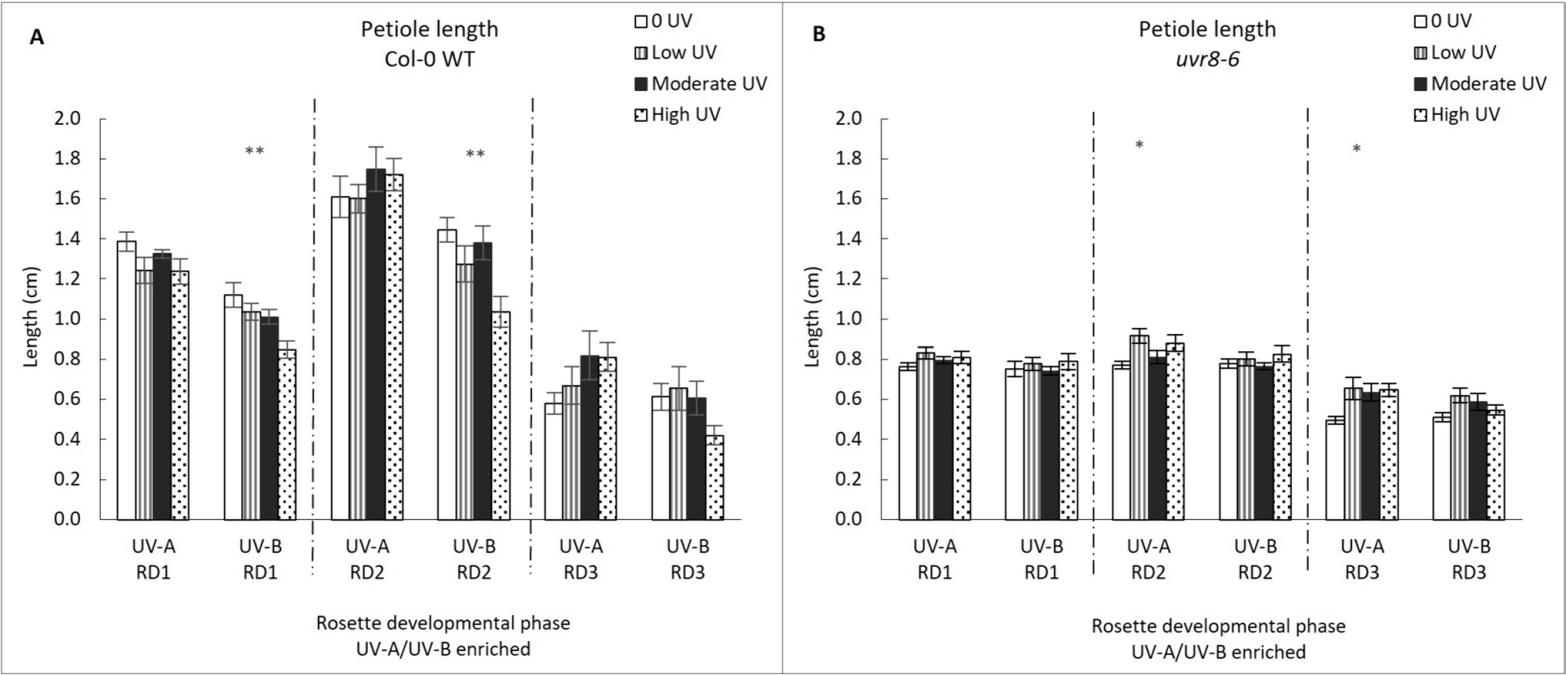
Petiole length (cm) in Col-0 WT (**A**) and *uvr8-6* (**B**), across the three rosette developmental phases (RD1, RD2, RD3) and two treatments (UV-A/B or UV-A enriched). Bar charts (with standard error) represent petiole averages. Bars represent different UV intensities: white bar = “0 UV”, striped bar = “Low UV”, black bar = “Moderate UV”, dotted bar = “High UV”. **A** UV-B significantly affected petioles of Col-0 in RD1 and RD2 (ANOVA, p = 0.003; K Wallis, p = 0.007). **B** Under UV-A petioles of *uvr8-6* were significantly different in RD2 and RD3 (K Wallis, p = 0.024; K Wallis, p = 0.016). Asterisks indicate a significant difference between 0 UV and low, moderate and high intensities with p < 0.05 (*), p < 0.01 (**), or p ≤ 0.001 (***)

The petiole response was dependent on intensity and UV treatment, and the presence of functional UVR8 photoreceptor (Fig. 4). Unlike leaf blade parameters, petioles of all developmental stages respond to UV treatment: developmental phase 1 (GLM: χ^2^ = 214.77, df = 9; p = < 0.001), phase 2 (GLM: χ^2^ = 232.63, df = 15; p = < 0.001) and phase 3 (GLM: χ^2^ = 27.107, df = 8; p = < 0.001). In phase 2, petiole length was primarily affected by UV treatment (p < 0.001) and genotype (p < 0.001), while interactive effects of intensity and genotype (p < 0.05), genotype and UV-treatment (p < 0.001) and UV-treatment and intensity (p < 0.05) also play a role. One-way ANOVA shows non-significant effects of the UV-A treatment on WT petiole length, but significant decreases in S1 and S2 petiole length with increasing intensity in the UV A/B enriched treatment in WT Arabidopsis (p = 0.003 and p = 0.007, respectively). In contrast, no changes in petiole length were observed in RD1, RD2 and RD3 of either UV-A or UV-A/B treated *uvr8-6* plants.

### Chlorophyll *a* fluorometry

**Fig. 5:**
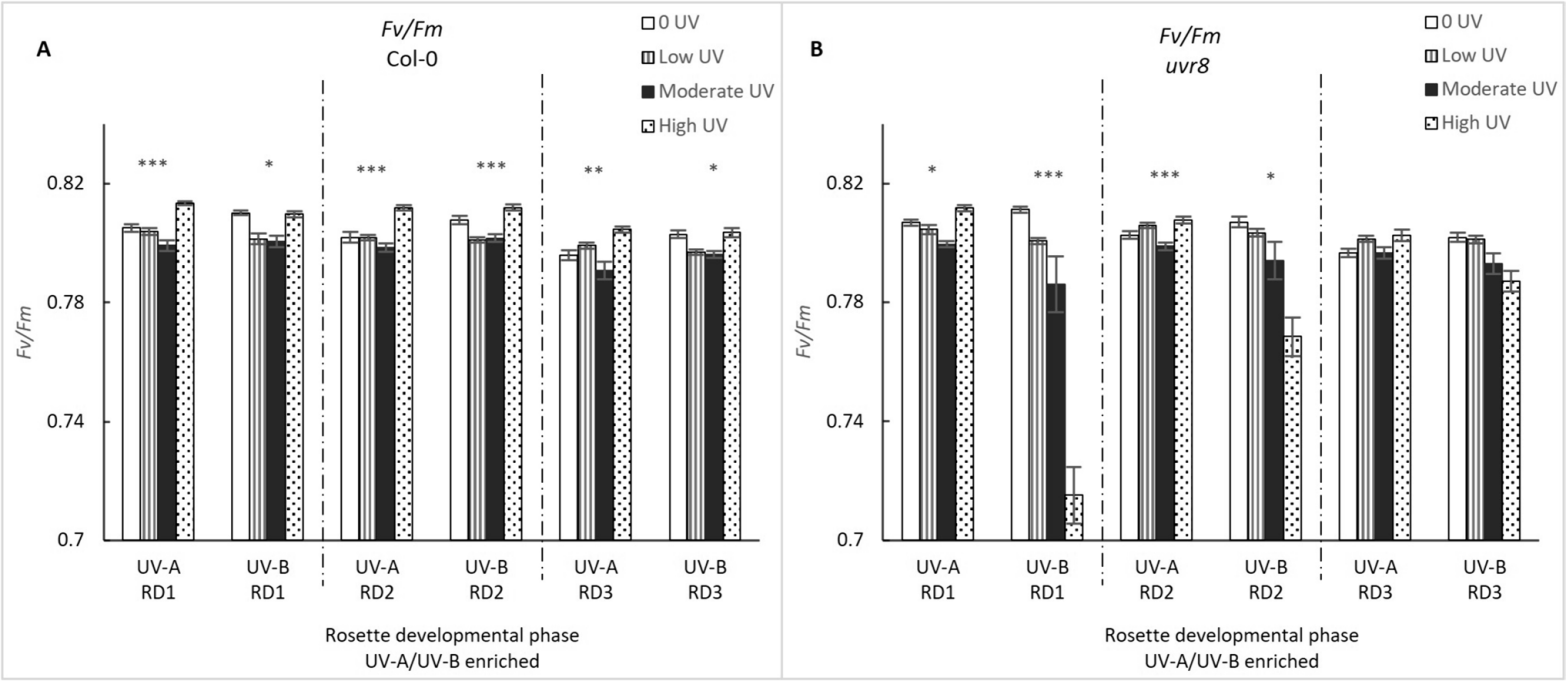
The maximum quantum yield of PSII, Fv/Fm in Col-0 (**A**) and *uvr8-6* (**B**) across the three rosette developmental phases (RD1, RD2, RD3) and two treatments (UV-A/B and UV-A enriched). Bars represent different UV intensities: white bar = “0 UV”, striped bar = “Low UV”, black bar = “Moderate UV”, dotted bar = “High UV”. **A** Across all developmental phases, UV-A had a significant effect on Fv/Fm values in Col-0 (K Wallis, p < 0.001; ANOVA, p < 0.001; Welch ANOVA, p = 0.01) as did UV-A/B (Welch ANOVA, p = 0.014; Welch, p < 0.001; K Wallis, p = 0.03) respectively. **B** In *uvr8-6* plants, effects were seen in RD1 and RD2 only for both UV-A (K Wallis, p = 0.019; ANOVA, p < 0.001) and UV-A/B (Welch ANOVA, p < 0.001; ANOVA, p = 0.02). Asterisks indicate a significant difference between 0 UV and low, moderate and high intensities with p < 0.05 (*), p < 0.01 (**), or p ≤ 0.001 (***)

The maximum quantum yield of photosystem II (PSII), Fv/Fm, was consistently high, varying between 0.79 and 0.81 for Col-0 WT and across all treatments (Fig. 5). For developmental phases 1 and 2, Fv/Fm values are significantly dependent on UV treatment, intensity, and the presence of functional UVR8 photoreceptor (phase 1 (GLM: χ^2^ = 207.55, df = 15; p < 0.0001), phase 2 (GLM: χ^2^ = 107.74, df = 15; p < 0.0001). However, effects are particularly small and most likely biologically not relevant, except for the statistical (ANOVA) decline in Fv/Fm values in the *uvr8* genotype exposed to the highest intensity of the UV-A/B enriched treatment, and across developmental phases 1 and 2 (p < 0.001 and p < 0.05, respectively).

**Fig. 6:**
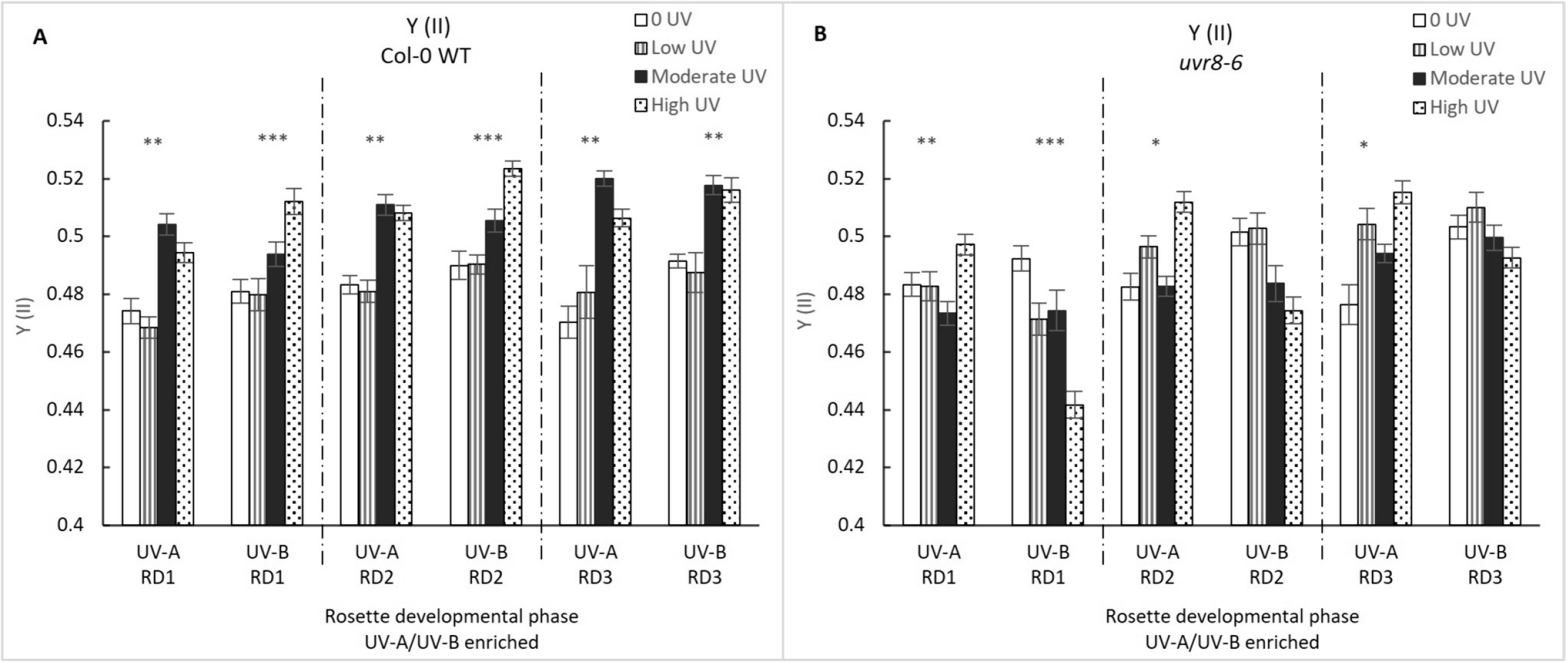
The effective quantum of PSII, Y(II), in Col-0 (**A**) and *uvr8-6* (**B**) across the three rosette developmental phases (RD1, RD2, RD3) and two treatments (UV-A/B and UV-A enriched). **A** Across all developmental phases, Bars represent different UV intensities: white bar = “0 UV”, striped bar = “Low UV”, black bar = “Moderate UV”, dotted bar = “High UV”. UV-A had a significant effect on Y(II) values in all phases of Col-0 plants (all Welch ANOVA, p < 0.01) as did UV-A/B (Welch ANOVA, p < 0.001 for RD1 and RD2; K Wallis, p < 0.001 in RD3). **B** In *uvr8-6* plants, UV-A significantly effected Y(II) across all phases (K Wallis, p = 0.006; K Wallis, p = 0.021; K Wallis, p = 0.030) while UV-A/B effected RD1 only (K Wallis, p < 0.001). Asterisks indicate a significant difference between 0 UV and low, moderate and high intensities with p < 0.05 (*), p < 0.01 (**), or p ≤ 0.001 (***)

The effective quantum yield of PSII under steady state conditions increased with increasing intensity across both UV-A and UV-A/B enriched treatments (Fig. 6). In this study, values of Y(II) were found to be relatively high and were associated with values of Y(NPQ) and Y(NO) of around 0.18 to 0.23 and 0.25 to 0.32, respectively. For developmental phases 1 and 2, Y(II) values are significantly dependent on intensity, genotype and interactive effects of genotype and intensity, intensity and treatment, intensity, genotype and UV-treatment (phase 1 (GLM: χ^2^ = 89.409, df = 15; p < 0.0001), phase 2 (GLM: χ^2^ = 88.266, df = 15; p < 0.0001). One-way ANOVA shows a significant increase in RD2 Y(II) with increasing intensity in the UV-A treatment in both WT and *uvr8-6* (p < 0.001 and p < 0.05 respectively). Similarly, a significant increase in Y(II) with increasing intensity is noted for WT phase 2 leaves exposed to the UV-A/B enriched treatment (p < 0.001 for both developmental phases 1 and 2). In contrast, a significant decrease in Y(II) is observed for *uvr8-6* phase SD1 leaves exposed to the UV-A/B treatment (p < 0.001), as well as a non-significant decrease in SD2 leaves.

### Total antioxidant capacity

**Fig. 7:**
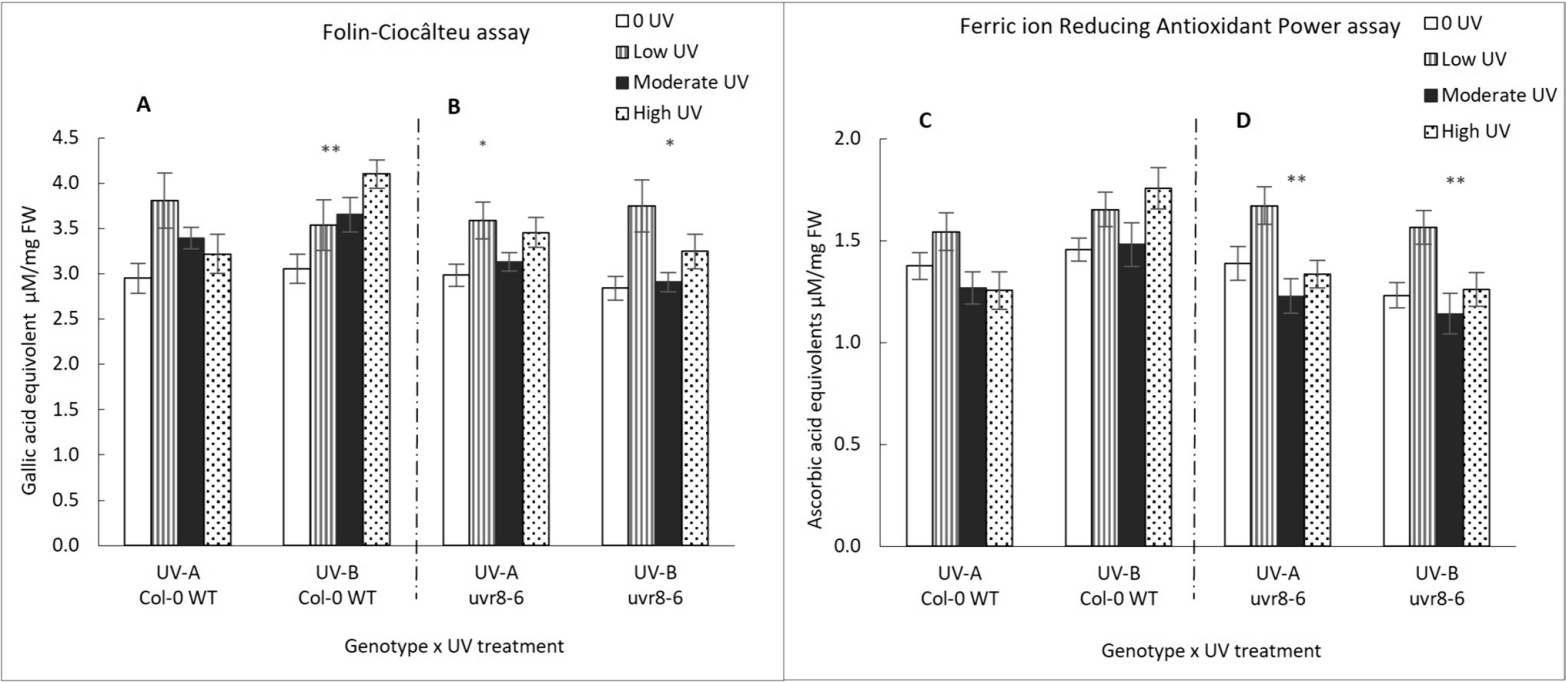
Concentration of total antioxidants for Col-0 WT (**A**) and *uvr8-6* (**B**), measured by the Folin Ciocâlteu assay and ferric ion reducing antioxidant power (FRAP) assay for Col-0 WT(**C**) and *uvr8-6* (**D**). Bar charts (with standard error) represent mean compound concentration in pooled leaves of whole rosettes across four intensities and two treatments (UV-A/B and UV-A enriched). Bars represent different UV intensities: white bar = “0 UV”, striped bar = “Low UV”, black bar = “Moderate UV”, dotted bar = “High UV”. **A** UV-A/B significantly affects concentrations in Col-0 (K Wallis, p = 0.004) and in *uvr8-6* plants (**B**) (K Wallis, p = 0.04). **B** UV-A had a significant effect on concentrations in *uvr8-6* (ANOVA, p = 0.03). **C** UV-A and UV-A/B treatments had a statistically significant effect on antioxidant capacity of *uvr8-6* (ANOVA, p = 0.005) and (ANOVA, p = 0.006) respectively. Asterisks indicate a significant difference between 0 UV and low, moderate and high intensities with p < 0.05 (*), p < 0.01 (**), or p ≤ 0.001 (***)

The Folin-Ciocâlteu assay shows antioxidant activities varying between 3 and 4 gallic acid equivalents (µM/mg) for Col-0 WT and *uvr8-6* across all treatments (Fig. 7). Activities appear variable, and overall, the GLM model does not reveal a significant effect across genotypes, intensity and treatment (GLM: χ^2^ = 1.0599, df = 6; p < 0.98), although removal of non-significant terms indicated a significant effect of intensity and of the interaction between intensity and genotype. One-way ANOVA shows significant increases in antioxidant capacity in UV-A/B treated WT (p = 0.004), possibly associated with accumulation of flavonoids. This increase in antioxidant capacity is not distinguishable in *uvr8-6* plants exposed to UV-A/B enriched light.

Antioxidant capacity measured using the Ferric ion Reducing Antioxidant Power (FRAP) assay varied significantly (GLM: χ^2^ = 49.666, df = 6; p < 0.0001), depending on genotype (p < 0.01), intensity (p < 0.0001), as well as an interaction between genotype and UV-treatment (p < 0.0001: Fig. 7). One-way ANOVA shows small, but significant, decreases in ascorbic acid equivalents in *uvr8-6* plants exposed to UV-A/B or UV-A enriched treatments (p =  < 0.01 and p =  < 0.01, respectively).

## Discussion

This study presents to the dose specific response of *Arabidopsis thaliana* wildtype and a UVR8 impaired mutant to increasing UV radiation. Commonly used UV-B tubes, such as the Philips TL12 and the Q-panel UV-B, also emit a substantial amount of UV-A radiation [11]. In the current study, it was investigated whether small amounts of accompanying UV-A will affect UV-B induced “dwarfing” effects on leaf morphology. For this study, leaves were grouped in developmental phases 1 (oldest leaves, fully formed prior to UV exposure), phase 2 (leaves visible prior to UV exposure, but expanding during UV exposure) and phase 3 (not visible prior to UV exposure). Effects of UV-A/B and UV-A enriched conditions on leaf blade expansion and leaf blade area could be observed across phases 2 and 3, but not 1. This most likely reflects the cessation of cell division and elongation in these older leaves [33]. In contrast, UV-effects on petiole elongation where still notable for leaves in phase 1, and this most likely relates to the basipetal direction of leaf maturation whereby regions near the tip of the leaf cease expanding first and those near the base last [33].

### Effects of UV-A on rosette development

Leaf area is an important determinant of photon capture, and hence photosynthesis, but also of gas-exchange and hence transpiration. Consequently, the development of leaf area is tightly controlled and this involves regulation of cell division, differentiation and expansion in response to various environmental factors (cf [34]). A well-known example of the environmental control of leaf area is the development of relatively small sun leaves in sun exposed positions on trees, compared to larger leaves inside the shady interior of the crown [35]. However, dose-responses appear somewhat more complex as in low light environments increases in PAR can result in increased leaf area [36]. The light spectrum is another important determinant of leaf expansion. Growth under blue light is known to result in increased leaf expansion [37]. Stimulatory effects of UV-A radiation on leaf elongation have also been reported [7, 38, 39], although these effects are not present in all species under all conditions [21]. These blue and UV-A induced effects on leaf expansion are thought to be mediated by phototropins [40] and cryptochromes [41]. Interestingly, the current study shows that UV-A mediated elongation is a rather specific process that affects some aspects of leaf expansion, but not others. For example, UV-A exposure leads to an increase in blade length but not blade width. Thus, UV-A mediates expansion along the proximal–distal axis of the blade, but not the medio-lateral axis. The resulting increase in blade length: width ratio has been predicted [42] to contribute to increased light capture per unit leaf area due to a reduced aggregation of leaf area around the stem. It could be surmised that petiole elongation would further increase light capture, however petiole length is not affected by UV-A exposure. This scenario is distinct from the classical shade avoidance response whereby leaf blade elongation is impeded, but petiole elongation is promoted, resulting in an increased petiole to leaf blade length ratio [43, 44]. Thus, the UV-A mediated elongation response is phenotypically distinct from the red/far-red mediated elongation response.

### Effects of UV-A plus UV-B on rosette development

Inhibitory effects of UV-B radiation on plant elongation responses have been reported by multiple authors, and this phenomenon is sometimes referred to as UV-B mediated dwarfing [19]. Although there is a broad consensus that exposure to UV-B can drive a relative compact plant architecture, phenotypes reported in the literature vary, and this may relate to (UV) exposure conditions and/or genotype. Indeed, the mechanism underlying the more compact architecture has been linked to a variety of possible mechanisms, including the activity of the UV-B photoreceptor UVR8, plant stress (i.e., stress-induced morphogenic responses) and UV-B induced metabolic reprofiling [19]. The current study shows that the UV-B nullifies the UV-A mediated increases in blade length and leaf area, while having a strong inhibitory effect on petiole length. It is likely that the inhibitory UV-B effect on petioles is particularly pronounced as there is no antagonistic (i.e., stimulating) UV-A effect on petioles. Inhibitory effects of UV-B radiation on petiole elongation can be observed in the WT but not *uvr8-6*. Thus, it is concluded that the effects on petiole length are mediated by the UV-B photoreceptor UVR8. Similarly, the inhibitory effects of UV-B on blade length and leaf area are UVR8 dependent. It has been argued that UV-B antagonises the classical shade avoidance response [45]. In such a shade-avoidance response leaf blade elongation is impeded, but petiole elongation is promoted [43]. This study shows that UV-B radiation, contrary to a low red to far-red (R to FR) light ratio, impedes petiole elongation, but similarly to a low R to FR ratio impedes leaf blade elongation. Thus, UV-B partly antagonises shade avoidance, and partly strengthens shade avoidance. Distinct UV-B effects on different aspects of leaf morphology are consistent with the existence of “separate and overlapping pathways” for shade avoidance, whereby elongation of different organs is controlled through distinct interactions between largely conserved signalling networks [46].

Intriguingly, a small but significant UV-B mediated decrease in leaf blade width is noted in developmental phase 2 of both WT and *uvr8-6* plants. This may represent an UVR8 independent effect. Previously, Wargent et al. [47] noted that epidermal cell division in response to UV-B is largely independent of UVR8. Other studies have reported UVR8 independent changes in gene-expression under very low UV intensities [48]. The putative UVR8 independent changes presented in this paper are accompanied by small declines in both Fv/Fm and Y(II) in *uvr8-6* plants exposed to the highest UV-B intensity. The slightly stronger decrease in Fv/Fm, compared to Y(II), most likely reflects the high UV sensitivity of photosystem II, relative to other, more rate limiting, aspects of the photosynthetic machinery [49]. The decrease in photosynthetic efficiency in *uvr8-6* is matched by a relative lack of upregulation of antioxidant defences (both gallic acid and ascorbic acid equivalents) in *uvr8-6* plants under the same UV conditions. These data imply a lack of UV-mediated accumulation of both flavanols and phenolic acids in *uvr8-6* and are consistent with a role of the UV-B photoreceptor in controlling biosynthesis of these compounds [17]. In contrast, antioxidant defences are upregulated in Col-0 plants exposed to the highest UV-A/B intensity, thus these plants display an acclimatory response that is absent in *uvr8-6* plants. The lack of this acclimative eustress response in uvr8-6 plants, together with the observed decrease in Y(II) implies that plants are subject to distress [6], and that the observed UVR8 independent morphological effects are stress-associated.

### Rosette morphology and the UV-B:UV-A ratio

The current study shows the distinct, and in some cases antagonistic effects, of UV-A and UV-B radiation on leaf morphology. Many commonly used broad band UV-B sources emit UV-B as well as UV-A and UV-C radiation [11]. The deleterious effects of UV-C have been extensively reported, and most studies employ long-pass cellulose acetate filters to remove such wavelengths form the exposure spectrum. However, many UV studies fail to remove UV-A wavelengths emitted by UV-B tubes. The current study shows that substantial impact of the accompanying UV-A wavelengths on morphological responses. As a result, it can be surmised that effects of UV-B are in some cases partly masked by effects of accompanying UV-A (e.g. leaf blade elongation). This leads to a potential underestimate of the magnitude of the UV-B response. The concurrent activation of UV-B and UV-A signalling pathways may also result in a failure to distinguish distinct UV-A and UV-B signalling elements in molecular studies. Conversely, with the discovery of crosstalk between UV-A and UV-B signalling cascades [8, 10], it cannot be excluded that the simultaneous exposure to UV-B and accompanying UV-A leads to completely new UV responses, and unexpected impacts on leaf morphology. The rapidly advancing development of wavelength-specific UV-LEDs, with a narrow output spectrum, will enable both accurate “UV-B only” studies, as well as light mixing experiments whereby specific UV-B and UV-A wavelengths are mixed.

The current study has shown the ameliorating effects accompanying UV-A wavelengths, emitted by broad band UV-B sources, on UV-B responses. In this study a relatively low intensity of UV-A radiation was found to modify several leaf morphological parameters. In contrast, the natural solar UV spectrum reaching the Earth’s surface is comprised for about 95% of the UV-A photons [11]. Thus, the question arises whether any effect of UV-B of leaf morphology can be discerned under UV-A enriched solar conditions, or rather whether such morphological effects will be masked in full spectrum experiments. A recent meta-analysis reveals UV-induced decreases in leaf area across multiple fields, glasshouse, and growth chamber experiments [50]. Therefore, perhaps the question is not whether UV-B effects can be discerned under UV-A rich sunlight, but rather, how it is possible that effects driven by low intensities of UV-B effects are still discernible under these conditions.

### A functional role for UV-mediated changes in leaf morphology

**Fig. 8:**
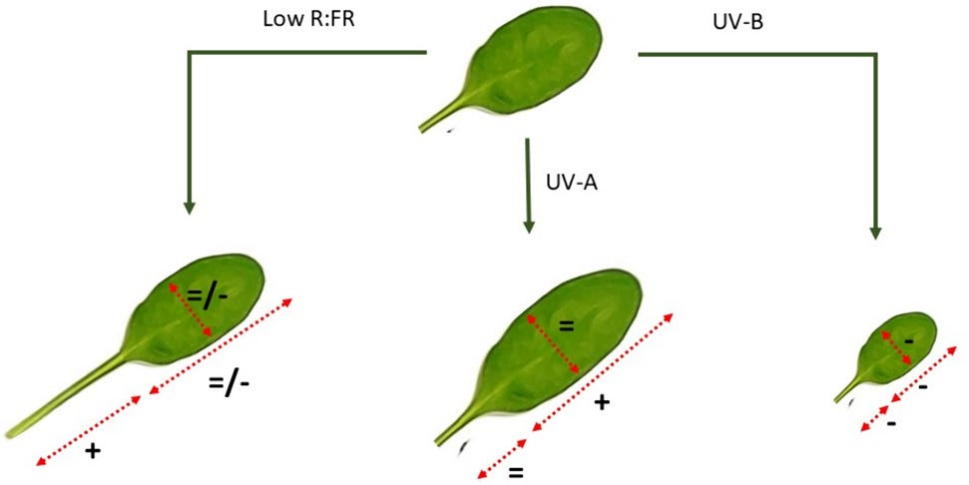
Schematic (cartoon-like) overview of distinct effects light on leaf morphology. Low R:FR represents classical shade avoidance under low red to far-red light mixtures and comprises strong petiole elongation [43, 44]. Data in this paper (Figs. 1, 2, 3, 4, 5) show that UV-A exposure drives an increase in leaf length, while UV-B exposure triggers shortening of petioles, blade length and blade width

Here, UV-B induced changes are reported in the context of distinct UV-A responses and compared to the shade avoidance response driven by low red to far-red ratios. In the latter case, the extension of petioles will directly decrease self-shading in rosettes around the stem, and this response has been modelled to enhance photosynthetic light capture [42] (Fig. 8). The measured UV-A driven increase in blade length: width ratio will similarly contribute to increased PAR capture per unit leaf area, with light capture further enhanced by an increase in leaf area (Fig. 8). Thus, UV-A mediated morphological responses can complement other mechanisms that increase light capture, such as potential increases in chlorophyll and carotenoid content. The response driven by UV-A (and presumably blue wavelengths) reinforces the low red to far-red response but will to some extent be distinct due to the increased importance of scattered light at shorter wavelengths [20]. In contrast, the measured UV-B mediated decrease in petiole elongation, together with a decrease in leaf area, will result in a decrease in both PAR and UV-B capture (Fig. 8). Yet, a key question remains whether this UV-B mediated change in leaf morphology serves to limit UV-B exposure as suggested in the past [12, 19]. At present any evidence for an avoidance strategy remains speculative and unsatisfactory, particularly given that UV-B damage to plants and crops is rare [6]. Therefore, the possibility that UV-induced acclimatory responses have a function other than UV-B protection, need to be considered. Recently, it has been noted that many of the morphological acclimation responses observed in UV-B exposed plants are similar to those contributing to drought resistance [50]. The compact UV-B phenotype with a reduced leaf area observed in this study is consistent with such a function.

## Data Availability

Data are available on request.

## References

[1] Heijde M, Ulm R (2012). UV-B photoreceptor-mediated signalling in plants. Trends in Plant Science.

[2] Paik I, Huq E (2019). Plant photoreceptors: Multi-functional sensory proteins and their signaling networks. Seminars in cell & developmental biology.

[3] Kilkenny FF, Galloway LF (2008). Reproductive success in varying light environments: Direct and indirect effects of light on plants and pollinators. Oecologia.

[4] Rizzini L, Favory JJ, Cloix C, Faggionato D, O’Hara A, Kaiserli E, Baumeister R, Schäfer E, Nagy F, Jenkins GI, Ulm R (2011). Perception of UV-B by the Arabidopsis UVR8 protein. Science.

[5] Rodríguez-Calzada T, Qian M, Strid Å, Neugart S, Schreiner M, Torres-Pacheco I, Guevara-González RG (2019). Effect of UV-B radiation on morphology, phenolic compound production, gene expression, and subsequent drought stress responses in chili pepper (*Capsicum annuum* L.). Plant Physiology and Biochemistry.

[6] Hideg É, Jansen MAK, Strid Å (2013). UV-B exposure, ROS, and stress: Inseparable companions or loosely linked associates?. Trends in Plant Science.

[7] Verdaguer D, Jansen MAK, Llorens L, Morales LO, Neugart S (2017). UV-A radiation effects on higher plants: Exploring the known unknown. Plant Science.

[8] Rai N, O'Hara A, Farkas D, Safronov O, Ratanasopa K, Wang F, Lindfors AV, Jenkins GI, Lehto T, Salojärvi J, Brosché M (2020). The photoreceptor UVR8 mediates the perception of both UV-B and UV-A wavelengths up to 350 nm of sunlight with responsivity moderated by cryptochromes. Plant, Cell & Environment.

[9] Díaz-Ramos LA, O'Hara A, Kanagarajan S, Farkas D, Strid Å, Jenkins GI (2018). Difference in the action spectra for UVR8 monomerisation and HY5 transcript accumulation in Arabidopsis. Photochemical & Photobiological Sciences.

[10] Rai N, Neugart S, Yan Y, Wang F, Siipola SM, Lindfors AV, Winkler JB, Albert A, Brosché M, Lehto T, Morales LO (2019). How do cryptochromes and UVR8 interact in natural and simulated sunlight?. Journal of Experimental Botany.

[11] Aphalo PJ, Albert A, Björn LO, McLeod AR, Robson TM, Rosenqvist E (2012). Beyond the visible: A handbook of best practice in plant UV photobiology.

[12] Jansen MAK, Gaba V, Greenberg BM (1998). Higher plants and UV-B radiation: Balancing damage, repair and acclimation. Trends in Plant Science.

[13] Paul N, Gwynn-Jones D (2003). Ecological roles of solar UV radiation: Towards an integrated approach. Trends in Ecology and Evolution.

[14] Shi C, Liu H (2021). How plants protect themselves from ultraviolet-B radiation stress. Plant Physiology.

[15] Mackerness SAH (2000). Plant responses to ultraviolet-B (UV-B: 280–320 nm) stress: What are the key regulators?. Plant Growth Regulators.

[16] Csepregi K, Neugart S, Schreiner M, Hideg É (2016). Comparative evaluation of total antioxidant capacities of plant polyphenols. Molecules.

[17] Jenkins GI (2014). The UV-B photoreceptor UVR8: From structure to physiology. The Plant Cell.

[18] Potters G, Pasternak TP, Guisez Y, Palme KJ, Jansen MAK (2007). Stress-induced morphogenic responses: Growing out of trouble?. Trends in Plant Science.

[19] Robson TM, Klem K, Urban O, Jansen MAK (2015). Re-interpreting plant morphological responses to UV-B radiation. Plant, Cell & Environment.

[20] Flint SD, Caldwell MM (1998). Solar UV-B and visible radiation in tropical forest gaps: Measurements partitioning direct and diffuse radiation. Global Change Biology.

[21] Qian M, Rosenqvist E, Prinsen E, Pescheck F, Flygare AM, Kalbina I, Jansen MAK, Strid Å (2021). Downsizing in plants—UV light induces pronounced morphological changes in the absence of stress. Plant Physiology.

[22] Brodführer U (1955). Der einfluss einer abgestuften dosierung von ultravioletter sonnenstrahlung auf das wachstum der pflanzen. Planta.

[23] Qaderi MM, Yeung EC, Reid DM (2008). Growth and physiological responses of an invasive alien species, *Silene noctiflora*, during two developmental stages to four levels of ultraviolet-B radiation. Ecoscience.

[24] Reddy KR, Singh SK, Koti S, Kakani VG, Zhao D, Gao W, Reddy VR (2013). Quantifying corn growth and physiological responses to ultraviolet-B radiation for modeling. Agronomy Journal.

[25] Favory JJ, Stec A, Gruber H, Rizzini L, Oravecz A, Funk M, Albert A, Cloix C, Jenkins GI, Oakeley EJ, Seidlitz HK (2009). Interaction of COP1 and UVR8 regulates UV-B-induced photomorphogenesis and stress acclimation in Arabidopsis. The EMBO Journal.

[26] Boyes DC, Zayed AM, Ascenzi R, McCaskill AJ, Hoffman NE, Davis KR, Görlach J (2001). Growth stage-based phenotypic analysis of Arabidopsis: A model for high throughput functional genomics in plants. The Plant Cell.

[27] Flint SD, Caldwell MM (2003). A biological spectral weighting function for ozone depletion research with higher plants. Physiologia Plantarum.

[28] Ferreira T, Rasband W (2012). ImageJ user guide. ImageJ/Fiji.

[29] Murchie EH, Lawson T (2013). Chlorophyll fluorescence analysis: A guide to good practice and understanding some new applications. Journal of Experimental Botany.

[30] Csepregi K, Kocsis M, Hideg É (2013). On the spectrophotometric determination of total phenolic and flavonoid contents. Acta Biologica Hungarica.

[31] Singleton VL, Rossi JA (1965). Colorimetry of total phenolics with phosphomolybdic–phosphotungstic acid reagents. American Journal of Enology and Viticulture.

[32] Benzie IFF, Strain JJ (1996). The ferric reducing ability of plasma (FRAP) as a measure of ‘antioxidant power’: The FRAP assay. Analytical Biochemistry.

[33] Thomas B, Thomas B, Murray BG, Murphy DJ (2017). Leaf development. Encyclopedia of applied plant sciences.

[34] Horiguchi G, Ferjani A, Fujikura U, Tsukaya H (2006). Coordination of cell proliferation and cell expansion in the control of leaf size in *Arabidopsis thaliana*. Journal of Plant Research.

[35] Mathur S, Jain L, Jajoo A (2018). Photosynthetic efficiency in sun and shade plants. Photosynthetica.

[36] Pawłowska B, Żupnik M, Szewczyk-Taranek B, Cioć M (2018). Impact of LED light sources on morphogenesis and levels of photosynthetic pigments in *Gerbera jamesonii* grown in vitro. Horticulture, Environment, and Biotechnology.

[37] Wang XY, Xu XM, Cui J (2015). The importance of blue light for leaf area expansion, development of photosynthetic apparatus, and chloroplast ultrastructure of *Cucumis sativus* grown under weak light. Photosynthetica.

[38] Kong Y, Zheng Y (2020). Phototropin is partly involved in blue-light-mediated stem elongation, flower initiation, and leaf expansion: A comparison of phenotypic responses between wild Arabidopsis and its phototropin mutants. Environmental and Experimental Botany.

[39] Zhang Y, Sun X, Aphalo PJ, Zhang Y, Cheng R, Li T (2023). Ultraviolet-A1 radiation induced a more favorable light-intercepting leaf-area display than blue light and promoted plant growth. Plant, Cell & Environment..

[40] Legris M, Szarzynska-Erden BM, Trevisan M, Allenbach Petrolati L, Fankhauser C (2021). Phototropin-mediated perception of light direction in leaves regulates blade flattening. Plant Physiology.

[41] Li QH, Yang HQ (2007). Cryptochrome signaling in plants. Photochemistry and Photobiology.

[42] Takenaka A (1994). Effects of leaf blade narrowness and petiole length on the light capture efficiency of a shoot. Ecological Research.

[43] Kozuka T, Horiguchi G, Kim GT, Ohgishi M, Sakai T, Tsukaya H (2005). The different growth responses of the *Arabidopsis thaliana* leaf blade and the petiole during shade avoidance are regulated by photoreceptors and sugar. Plant and Cell Physiology.

[44] Sng BJR, Singh GP, Van Vu K, Chua NH, Ram RJ, Jang IC (2020). Rapid metabolite response in leaf blade and petiole as a marker for shade avoidance syndrome. Plant Methods.

[45] Hayes S, Velanis CN, Jenkins GI, Franklin KA (2014). UV-B detected by the UVR8 photoreceptor antagonizes auxin signaling and plant shade avoidance. Proceedings of the National Academy of Sciences.

[46] Nozue K, Tat AV, Kumar Devisetty U, Robinson M, Mumbach MR, Ichihashi Y, Lekkala S, Maloof JN (2015). Shade avoidance components and pathways in adult plants revealed by phenotypic profiling. PLoS Genetics.

[47] Wargent JJ, Gegas VC, Jenkins GI, Doonan JH, Paul ND (2009). UVR8 in *Arabidopsis thaliana* regulates multiple aspects of cellular differentiation during leaf development in response to ultraviolet B radiation. New Phytologist.

[48] O’Hara A, Headland LR, Díaz-Ramos LA, Morales LO, Strid Å, Jenkins GI (2019). Regulation of Arabidopsis gene expression by low fluence rate UV-B independently of UVR8 and stress signaling. Photochemical & Photobiological Sciences.

[49] Roháček K, Soukupová J, Barták M (2008). Chlorophyll fluorescence: a wonderful tool to study plant physiology and plant stress. Plant Cell Compartments-Selected Topics. Research Signpost, Kerala, India.

[50] Jansen MAK, Ač A, Klem K, Urban O (2022). A meta-analysis of the interactive effects of UV and drought on plants. Plant, Cell & Environment.

